# Mitochondrion genomes of seven species of the endangered genus ****Sporophila**** (Passeriformes: Thraupidae)

**DOI:** 10.1590/1678-4685-GMB-2023-0172

**Published:** 2024-04-05

**Authors:** Amanda Alves de Melo-Ximenes, Leonardo Carlos Jeronimo Corvalán, Larissa Resende Carvalho, Thalita Alves Mangini, Mariane Brom Sobreiro, Lucas Donizetti Vieira, Renata de Oliveira Dias, Carlos de Melo e Silva, Mariana Pires de Campos Telles, Rhewter Nunes

**Affiliations:** 1 Universidade Federal de Goiás, Instituto de Ciências Biológicas (ICB), Laboratório de Genética & Biodiversidade (LGBio), Goiânia, GO, Brazil.; 2 Universidade Federal de Goiás, Instituto de Ciências Biológicas (ICB), Programa de Pós-Graduação em Genética e Biologia Molecular, Goiânia, GO, Brazil.; 3 Universidade Estadual de Goiás, Instituto Acadêmico de Ciências da Saúde e Biológicas (IACSB), Laboratório de Bioinformática e Biodiversidade (LBB), Campus Oeste, Unidade Universitária de Iporá, Iporá, GO, Brazil.; 4 Instituto Tecnológico Vale Desenvolvimento Sustentável, Belém, PA, Brazil.; 5 Instituto Federal de Goiás, Goiás, GO, Brazil.; 6 Pontifícia Universidade Católica de Goiás, Escola de Ciências Médicas e da Vida, Goiânia, GO, Brazil.

**Keywords:** Aves, birds, mtDNA, mitogenome, phylogeny

## Abstract

We announce the mitochondrial genomes of seven species of the genus *Sporophila* (*S. bouvreuil, S. iberaensis, S. melanogaster, S. minuta, S. nigrorufa, S. pileata,* and *S. ruficollis*) which were validated by comparative genomic and phylogenetic analysis with related species. The mitochondrial genomes of seven passerines of the genus *Sporophila* were assembled (three complete and four nearly complete genomes) and were validated by reconstructing phylogenetic relations within Thraupidae. The complete mitogenomes ranged from 16,781 bp in *S. ruficollis* to 16,791 bp in *S. minuta*. We identified a conserved genome composition within all mitogenomes with 13 protein-coding genes, 22 tRNAs and two rRNAs. We observed a bias in the nucleotide composition and six mutational hotspots in *Sporophila* mitogenomes. Our mitogenome-based phylogenetic tree has *S. minuta*, *S. maximiliani* and *S. nigricollis* as sister species of the remaining species in the genus. We present new mitogenome sequences for seven *Sporophila* species, providing new genomic resources that may be useful for research on the evolution, comparative genetics, and conservation of this threatened group.

The genus *Sporophila* (Passeriformes, Thraupidae) includes a number of threatened species and ranks among Brazil’s most illegally traded wildlife (Charity and Ferreira, 2020). Taxonomic identification of these species remains challenging due to similar morphological characteristics, especially females that share the same light brown plumage pattern (Campagna et al., 2010; Burns et al., 2014). Therefore, the usage of a barcode region for molecular identification of *Sporophila* species can be useful.

However, the standard animal molecular marker, *cytochrome c oxidase I* (COI), is not the best choice for groups with closely related species and recent diversification of their lineages, since they have not had enough time to accumulate differences and evolutionary changes in their genome sequences, as in the case of *Sporophila* (Campagna et al., 2010; Burns et al., 2014)*.* An alternative barcode region or the use of whole mitogenome sequences can be applied in such cases to better compare and molecularly differentiate species.

The use of whole mitogenome sequences can be a strategy to reduce taxonomic misidentifications and to increase the amount of publicly available data that can be used for evolutionary and population genetic studies, as well as for conservation purposes such as the development of molecular markers for species identification. The underutilized data contained in databases such as the National Center for Biotechnology Information (NCBI) provide valuable new genomic resources and information on species at risk of extinction. Therefore, the main goal of this work is to assemble and characterize new mitogenomes from *Sporophila* species, as well as to compare them and their phylogenetic relationships within the genus and the family Thraupidae.

Raw sequencing reads were obtained from the SRA database at NCBI under the accession number SRP103901 from the project PRJNA382416, where they were generated for assembly of a reference genome for *Sporophila hypoxantha* and for population-level resequencing of several *Sporophila* species (Campagna et al., 2017). Table S1 lists the species used in our study for which genome assembly failed, partially assembled, and completely assembled.

Data were downloaded using the fastq-dump tool from the SRA Toolkit v. 3.0.0 (https://trace.ncbi.nlm.nih.gov/Traces/sra/sra.cgi?view=software). We selected three whole genome sequence libraries with the largest amount of data for each *Sporophila* species. For genome assembly, we used the three largest genomic libraries for each species and additionally, one genomic library was created by concatenating the previous three libraries. For each of the four libraries we assembled the mitochondrial genome using: I) a seed sequence only or II) a seed with a reference genome, both using NovoPlasty v. 4.3.1 (Dierckxsens et al., 2017) (Table S1). These different strategies were tested in order to increase the number of successful or partially successful assemblies due to the low depth of sequencing coverage of some genomic libraries.

For the seed-based assembly, one of the three genes: COI (NC_035673.1:5404-6954), CYTB (NC_035673.1:13677-14819) or ND2 (NC_035673.1:4005-5044) from the mitochondrial genome of *S. maximiliani* was used (Ludwig et al., 2017), as well as a species-specific seed (COI(s)) based on COI sequences obtained from the NCBI Nucleotide Database (Table S1). The reference-based assembly used the *S. maximiliani* mitochondrial genome (NC_035673.1) as reference. In total, we tested 8 assemblies per library (I.a. Seed (COI); I.b. Seed (cytB); I.c. Seed (ND2); I.d. Seed (COI(s)); II.a. Seed (COI) + reference; II.b. Seed (cytB) + reference; II.c. Seed (ND2) + reference; II.d. Seed (COI(s)) + reference), for a maximum of 32 assemblies per species. 

For annotation, we prioritized the largest assembly using a single library. The assembled genomes were aligned to the reference sequences of *S. maximiliani* (NC_035673.1) and *S. hypoxantha* (NC_051465.1) and the annotation was performed using MITOS WebServer v. 2 (http://mitos2.bioinf.uni-leipzig.de/index.py). All sequences from the annotated features were individually aligned against the two reference genomes using MAFFT v. 7 (https://mafft.cbrc.jp/alignment/server/) and checked for start and stop codons within the protein-coding genes and for start and end positions for the remaining features. 

For the comparative and evolutionary analysis, we used the mitochondrial genomes (partial or complete) of 10 species of the genus *Sporophila*, seven of which were obtained in this work: *S. iberaensis*, *S. melanogaster*, *S. minuta*, *S. nigrorufa*, *S. pileata*, *S. ruficollis*, *S. hypoxantha* (NC_051465.1, Campagna et al., 2017), *S. nigricollis* (NC_071761, Morais et al., 2022) and *S. maximiliani* (NC_035673.1, Ludwig et al., 2017). For these species, the presence of bias in nucleotide composition was estimated using AT-skew ((A - T)/(A+T)) and GC-skew ((G - C)/(G+C)) (Perna and Kocher, 1995).

Genomic similarity and collinearity were determined using progressive alignment in Mauve v. 2.4.0 (Darling et al., 2004). The presence of rearrangement and inversion events on these genomes was also checked on Mauve. For the same nine species, the Relative Synonymous Codon Usage (RSCU) was calculated on MEGA11 v. 11 (Tamura et al., 2021) using the vertebrate mitochondrial genetic code.

For the comparative analysis, beyond the mitogenomes newly assembled in this study, we retrieved from the NCBI Genome Database the complete record and coding sequences of all the 13 Thraupidae species with assembled mitogenomes deposited there. For all protein-coding genes, the synonymous (*Ks*) and nonsynonymous (*Ka*) substitution rate, *Ka/Ks* ratio, and nucleotide diversity (π) for the entire sequence of the mitogenomes were estimated using DnaSP v. 6.12.0 (Rozas et al., 2017).

We reconstructed the phylogeny of several Thraupidae mitogenomes using a maximum likelihood tree with IQ-TREE v. 2.2.0 (Nguyen and Ho, 2016), using ModelFinder Plus (Kalyaanamoorthy et al., 2017) to determine the best-fitting nucleotide model (GTR+F+I+G4) and 1,000 bootstrap replicates. The species *Cardinalis cardinalis* and *Piranga ludoviciana* from the family Cardinalidae were used as outgroups. The resulting phylogenetic tree was plotted using FigTree v. 1.4.4 (http://tree.bio.ed.ac.uk/software/figtree/).

Of the 11 species for which we attempted mitochondrial genome assembly, seven were successful (Table S1). Three species had their complete mitogenome assembled using a single library, and four had their partial mitogenome assembled using either one or three combined libraries (Table S1). The strategy of using *S. maximiliani* data as seed and as a reference genome showed the highest assembly success rates among the assembled genomes. In contrast to most other assemblers, NovoPlasty does not try to assemble every single read, but rather extends the given seed until a circular genome has been formed (Dierckxsens et al., 2017) (Table S1).

The mitogenome size of the analyzed *Sporophila* species ranged from 14,543bp for *S. pileata* to 16,791bp for *S. minuta* (Table 1, Figure 1). The complete mitogenomes assembled here have a very conserved structure, showing the same number of genes (13 CDS, 22 tRNAs, and two rRNAs) and in the same order as previously described in other *Sporophila* species (Table 1) (Ludwig et al., 2017; Lima-Rezende et al., 2019; Morais et al., 2022). This highly conserved structure is consistent with previous observations for the avian class, which has been described as the class with the lowest rearrangement rate among animal mtDNA (Montaña-Lozano et al., 2022). Consistent with previous results, our progressive alignment on Mauve did not reveal the presence of genomic rearrangements, but instead identified a unique similarity block, suggesting that these species share highly similar genomic collinearity (Figure S1). The remaining four partial mitogenomes showed a similar structure but lacked the first and last tRNAs (*S. pileata* and *S. iberaensis*) or only the last tRNA (*S. melanogaster*, and *S. nigrorufa*) together with the control region (Table 1).

**Table 1 -:** Characterization of seven newly assembled, completely or partially, mitochondrial genomes from *Sporophila* species, together with three previously published mitogenomes of the same genus. Genome length and size of control regions are given in base pairs (bp). *Species with complete mitochondrial genome sequences.

Species	Genome length (bp)	Total genes	CDS genes	tRNA genes	rRNA genes	Control region (bp)	Accession number	Reference
S. bouvreuil*	16,781	37	13	22	2	1,199	BK062957	This work
S. hypoxantha*	16,778	37	13	22	2	1,213	NC_051465.1	Campagna et al. (2017)
*S. iberaensis*	14,617	35	13	20	2	-	BK062958	This work
S. maximiliani*	16,801	37	13	22	2	1,203	NC_035673.1	Ludwig et al. (2017)
S. nigricollis*	16,777	37	13	22	2	1,188	NC_071761	Morais et al. (2022)
*S. melanogaster*	14,664	36	13	21	2	-	BK062959	This work
S. minuta*	16,791	37	13	22	2	1,199	BK062960	This work
*S. nigrorufa*	14,643	36	13	21	2	-	BK062961	This work
*S. pileata*	14,543	35	13	20	2	-	BK062962	This work
S.ruficollis*	16,781	37	13	22	2	1,198	BK062963	This work

**Figure 1 -:**
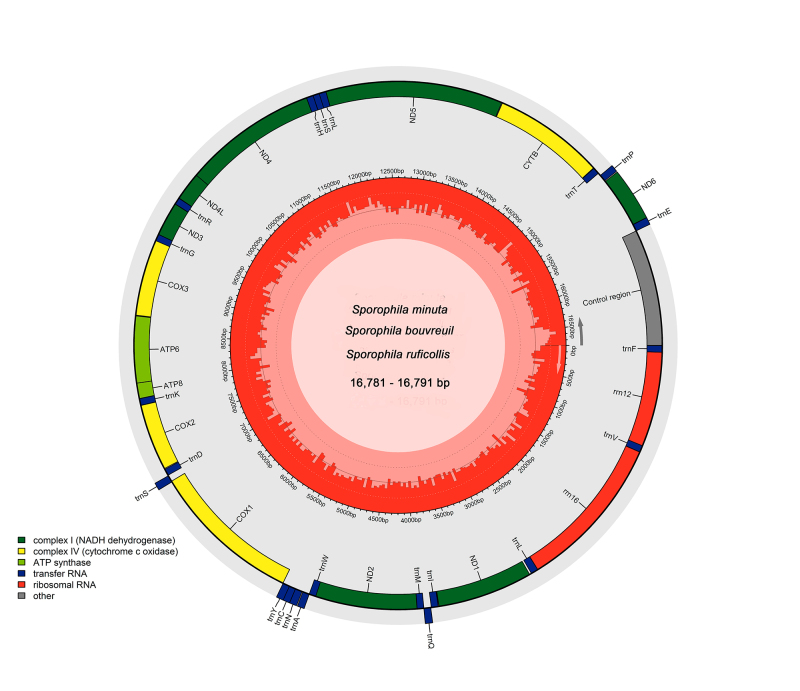
Graphical representation of the complete mitogenomes of three species of *Sporophila*: *S. minuta, S. bouvreuil,* and *S. ruficollis.* The mitogenomes showed identical structure and organization and are represented here together, with size ranging from 16,781 bp (*S. ruficollis* and *S. bouvreuil*) to 16,791 (*S. minuta*). Genes are colored indicated according to their functional classes, GC content is shown by the red bars inside the middle circle and reference position is indicated in base pairs (bp). Partially assembled mitogenomes of *S. melanogaster, S. iberaensis, S. nigrorufa*, and *S. pileata* are not presented in this representation due to missing features (tRNAs and control region).

The average GC content of the *Sporophila* mitochondrial genome was 46.95% (SD = 0.08) (Table S2). For all analyzed mitochondrial genomes, their complete sequence, PCG (Protein-coding genes) and rRNA showed a negative GC-skew and a positive AT-skew (Table S2). These values indicate a predominance of C over G and A over T, as previously described for *S. nigricollis* (Morais et al., 2022). The lower standard deviation value for GC content, GC-skew and AT-skew indicates a relative conservation of mitochondrial genome base composition in the genus *Sporophila*.

Among the nine mitochondrial genomes of the genus *Sporophila*, the number of codons ranged from 3,736 (*S. nigrorufa*) to 3,798 (*S. minuta*) (Figure S2). The most abundant codons for all *Sporophila* species were CUA (leucine) and AUC (isoleucine), while the most abundant amino acids were leucine, threonine and alanine. The RSCU analyses revealed preferential codon usage (Figure S2, Table S3), in agreement with other Thraupidae species, such as one of Darwin’s finches, *Geospiza magnirostris* (Xu et al., 2022). All tRNAs of the newly assembled *Sporophila* species showed a coverleaf-like structure (Figures S3 - S9).

The values of non-synonymous (*Ka*) and synonymous (*Ks*) substitutions for the 13 protein-coding genes of the 19 Thraupidae species ranged from 0 to 0.1225 for *Ka*, with a mean of 0.0303, and from 0 to 0.9254 for *Ks*, with a mean of 0.4169 (Figure 2A). The *ATP8* gene, which encodes a subunit of mitochondrial ATP synthase, had the highest *Ka* values (0.0686), followed by the *ND2* gene (0.0470), which encodes a subunit of mitochondrial NADH dehydrogenase (Figure 2A ). For *Ks* values, the *ND1* gene showed the highest values (0.6038), followed by the *ND2* gene (0.5450), and both genes encode subunits of mitochondrial NADH dehydrogenase synthase. 

**Figure 2 -:**
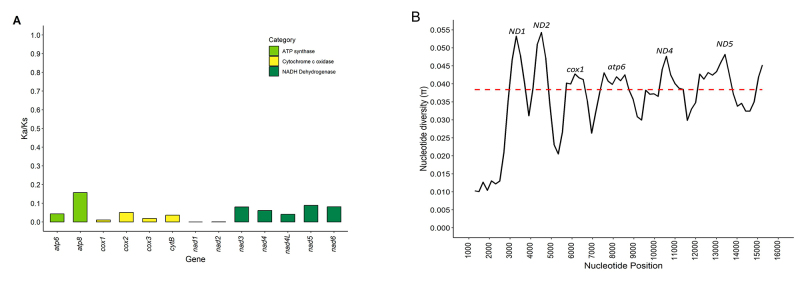
A) Rates of *Ka/Ks* for each of the 13 protein coding genes estimated for all Thraupidae species. B) Nucleotide diversity (π) calculated for all *Sporophila* species with mitochondrial genomes available in the NCBI Genome database. The red dashed line represents the median nucleotide diversity and peaks above it represent nucleotide diversity hotspots for the group.

The *Ka/Ks* ratio was also estimated to detect potential selection signatures on these genes. Among the 13 protein-coding genes, all genes had values of *Ka/Ks* ratio <1 (Figure 2A). *Ka/Ks* ratio < 1 indicates that these genes are potentially under negative selection, with most of the observed variation resulting from synonymous substitutions, as seen in other passerine mitogenomes (Xu et al., 2022). The *Ka/Ks* ratio ranged from 0.0001 (*nad1*) to 0.1568 (*atp8*), with the *ATP8* gene having the highest *Ka/Ks* ratio (0.1568), followed by *ND5* (0.0889) (Figure 2A ). The higher accumulation of non-synonymous substitutions in the *ATP8* gene has also been reported in other avian species, such as the greater scaup and the pin-tailed snipe (Hu et al., 2017; Xu *et al.*, 2022).

Among the assembled mitogenomes, the nucleotide diversity ranged from 0.007 to 0.043 (Table S4). The NADH dehydrogenase genes, especially *ND1*, *ND2* and *ND5*, showed the highest nucleotide diversity values (Figure 2B ). Considering the high values of synonymous mutation rate in these genes, it seems that most of the accumulated diversity is of synonymous type and does not result in amino acid exchange.

Campagna et al. (2010) discussed the difficulty of separating *Sporophila* groups in South America using only DNA barcode markers, in addition to the lack of monophyly of the group. The barcode region of the *COI* gene has been shown to have higher intraspecific diversity and no barcode gap in *Sporophila* (Campagna *et al.*, 2010)*,* although it is widely used for molecular identification of birds (de Melo et al., 2021). High nucleotide diversity and the presence of a barcoding gap, *i.e.*, a gap between intra- and interspecific differences in nucleotide sequences, are expected characteristics of a good barcode marker (Hebert et al., 2003), which is not the case of *COI* for *Sporophila* (Campagna *et al.*, 2010). The *ND2* gene is already a commonly used region for molecular identification of Thraupidae species (Burns et al., 2014) and may be a better option for discriminating *Sporophila* than *COI* due to the higher nucleotide diversity within this group (Figure 2B). Therefore, one strategy to reduce these taxonomic assignment problems may be the use of whole mtDNA.

Here we present the first phylogeny using complete Thraupidae mtDNA with more than three *Sporophila* species (Figure 3). Although Thraupidae is one of the largest families of Passeriformes, only 13 species had mitochondrial genomes available on NCBI prior to this work. This underrepresentation status of molecular data highlights the urgent need for more genomic studies for the entire order given its size, diversity, and economic and biological importance (de Melo et al., 2021). In the most important work with the family Thraupidae, Burns et al. (2014), who evaluated 32 species (out of a total of 39 species so far) using different genomic regions (*cytb, ND2, ACO1-I9, MBI2, RAG1* gene), indicate a monophyletic group formed by the three genera *Oryzoborus, Sporophila* and *Dolospingus*. The authors reiterate that in addition to molecular markers, the region of occurrence, feeding behavior such as a granivorous diet, and morphology (body size, beak shape and color) also supports this group (Mason and Burns, 2013), and the three genera should be defined as *Sporophila*.

**Figure 3 -:**
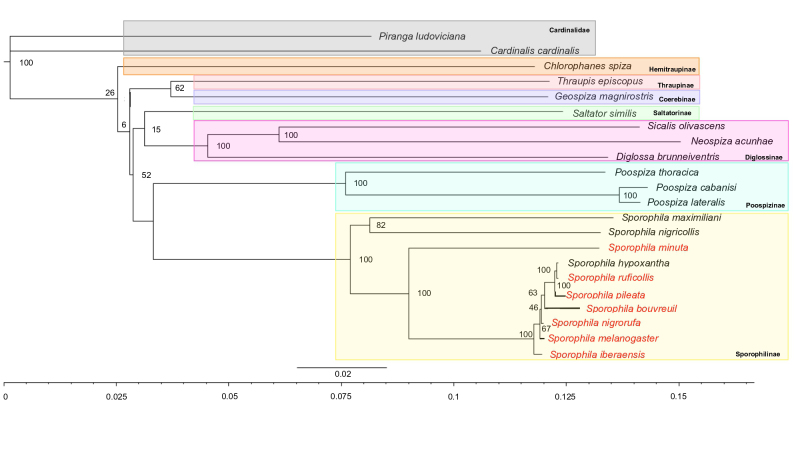
Mitogenome-based phylogenetic tree of Thraupidae family obtained using ML method. The values on the nodes represent bootstrap support from 1,000 replicates. Labels in red represent species whose mitogenomes were assembled in this work. Species are highlighted in different colors according to their subfamilies, which are written in bold.

Our study shows similar results to those of Lijtmaer et al. (2004) using other molecular and morphological markers (similar to the consensus tree of the cytochrome b gene and COII-Tlys-ATP8 fragments). The species *S. minuta*, *S. hypoxantha, S. ruficollis* and *S. melanogaster* all belong to group G (capuchin group), originally proposed by Ridgely and Tudor (1994). Within this group, the species still differ enough to be subdivided into internal groups, such as *S. maximiliani*, *S. nigricollis* and *S. minuta*, which form a more closely related group (Figure 3). For *S. iberaensis*, this is the first time that this species has appeared on a phylogenetic tree with molecular data, either for the genus or for the family, and it has been placed more closely related to *S. melanogaster* and *S. nigrorufa* (Figure 3). The resulting topology of the relationship between *Sporophila* species and other Thraupidae species, including subfamily relationships, was similar to previous studies that placed all *Sporophila* species in a single clade (Campagna et al., 2010; Burns et al., 2014) (Figure 3) and validated the assembly of the new mitogenomes.

This work provides novel mitogenome sequences for the genus *Sporophila*, which showed a similar structure to other species of the genus and of the Thraupidae family. The mitogenomes analyzed here show a conserved pattern, which is evidenced by the maintenance of gene order, low nucleotide diversity and signs of negative selection. Furthermore, the seven *Sporophila* species were consistently gathered in a clade with other Thraupidae species in a phylogenetic analysis. The provided mtDNA sequences can help elucidate taxonomic relationships unclear within the group and be useful to several studies involving these endangered species.

## Supplementary material

The following online material is available for this article:

Table S1 **-**
Results of mitochondrial genome assembly strategy using NOVOplasty v4.3.1.

Table S2 - Nucleotide composition bias and GC content for ten *Sporophila* mitochondrial genome.

Table S3 - Number of codons and Relative Synonymous Codon Usage (RSCU) for ten *Sporophila* mitochondrial genomes.

Table S4 - Nucleotide diversity of *Sporophila* species mitochondrial genomes.

Figure S1 - Progressive alignment performed on MAUVE showing a unique similarity block between *Sporophila* species shown in the respective order*.*

Figure S2 - Relative Synonymous Codon Usage (RSCU) analysis for all nine *Sporophila* species with complete mitochondrial genome currently available.

Figure S3 - Cover leaf-like structure of the 22 tRNAs present in the mitochondrial genome of *Sporophila bouvreuil*.

Figure S4 - Cover leaf-like structure of the 20 tRNAs present in the mitochondrial genome of *Sporophila iberaensis.*

Figure S5 - Cover leaf-like structure of the 21 tRNAs present in the mitochondrial genome of *Sporophila melanogaster.*

Figure S6 - Cover leaf-like structure of the 22 tRNAs present in the mitochondrial genome of *Sporophila minuta.*

Figure S7 - Cover leaf-like structure of the 21 tRNAs present in the mitochondrial genome of *Sporophila nigrorufa.*

Figure S8 - Cover leaf-like structure of the 20 tRNAs present in the mitochondrial genome of *Sporophila pileata.*

Figure S9 - Cover leaf-like structure of the 22 tRNAs present in the mitochondrial genome of *Sporophila ruficollis.*
