# Supplementary material for: Mitochondrion genomes of seven species of the endangered genus Sporophila (Passeriformes: Thraupidae)
Source: Genet Mol Biol. 2024 Apr 5;47(1):e20230172. doi: 10.1590/1678-4685-GMB-2023-0172 (PMC10995768; doi:10.1590/1678-4685-GMB-2023-0172)
Supplement: Table S1 - [file 1415-4757-GMB-47-1-e20230172-s1.pdf]

Genetics and Molecular Biology
https://doi.org/10.1590/1678-4685-GMB-2023-0172

Supplementary Material to "Mitochondrion genomes of seven species of the endangered genus *Sporophila* (Passeriformes: Thraupidae)"

Table S1 - Results of mitochondrial genome assembly strategy using NOVOplasty v4.3.1. Mitogenomes were considered successfully assembled if they presented length >16,000 bp, partially assembled if > 14,000 bp, and assembly failed if < 10,000 pb. LC: Least concerned; VU: Vulnerable; EN: endangered; NT: Near Threatened. NA: Not available.

Species	Threat level	SRA NCBI ID	Assembly strategy								COI(s) accession number
			COI seed	*Cytb seed*	*ND2 seed*	COI + reference	Cytb + reference	ND2 + reference	COI(s)	COI(s) + reference	
Sporophila bouvreuil	LC	SRR14546107	Success	Success	Success	Success	Success	Success	Success	Success	KF316358.1
Sporophila castaneiventris	LC	-	Assembly failure	Assembly failure	Assembly failure	Assembly failure	Assembly failure	Assembly failure	Assembly failure	Assembly failure	GU070583.1
Sporophila cinnamomea	VU	-	Assembly failure	Assembly failure	Assembly failure	Assembly failure	Assembly failure	Assembly failure	Assembly failure	Assembly failure	FJ028304.1
Sporophila hypochroma	NT	-	Assembly failure	Assembly failure	Assembly failure	Assembly failure	Assembly failure	Assembly failure	Assembly failure	Assembly failure	FJ028311.1
Sporophila iberaensis	EN	SRR12775038	Partial	Partial	Partial	Partial	Partial	Partial	Assembly failure	NA	NA
Sporophila melanogaster	NT	SRR5447374 SRR5447381 SRR5447373	Partial	Partial	Partial	Partial	Partial	Partial	Partial	Partial	GU070605.1
Sporophila minuta	LC	SRR14546109	Success	Success	Success	Success	Success	Success	Success	Success	GU070592.1
Sporophila nigrorufa	VU	SRR5447346 SRR5447350 SRR5447348	Partial	Assembly failure	Partial	Partial	Partial	Partial	Partial	Partial	GU070612.1
Sporophila palustris	EN	-	Assembly failure	Assembly failure	Assembly failure	Assembly failure	Assembly failure	Assembly failure	Assembly failure	Assembly failure	KF316400.1
Sporophila pileata	LC	SRR5447396 SRR5447395 SRR5447394	Assembly failure	Partial	Assembly failure	Assembly failure	Partial	Assembly failure	Assembly failure	Assembly failure	KF316355.1
Sporophila ruficollis	NT	SRR14554186	Success	Success	Success	Success	Success	Success	Success	Success	FJ028320.1
