# Supplementary material for: Mitochondrion genomes of seven species of the endangered genus Sporophila (Passeriformes: Thraupidae)
Source: Genet Mol Biol. 2024 Apr 5;47(1):e20230172. doi: 10.1590/1678-4685-GMB-2023-0172 (PMC10995768; doi:10.1590/1678-4685-GMB-2023-0172)
Supplement: Table S2 - [file 1415-4757-GMB-47-1-e20230172-s2.pdf]

Genetics and Molecular Biology
https://doi.org/10.1590/1678-4685-GMB-2023-0172

Supplementary Material to "Mitochondrion genomes of seven species of the endangered genus *Sporophila* (Passeriformes: Thraupidae)"

Table S2 - Nucleotide composition bias and GC content for ten *Sporophila* mitochondrial genome. GC%: Percentage of guanine and cytosine; PCG: Protein coding gene; tRNA genes: transfer RNA genes; rRNA genes: ribosomal RNA genes.

Species	Genome			PCG			tRNA genes			rRNA genes		
	GC%	GC-skew	AT-skew	GC%	GC-skew	AT-skew	GC%	GC-skew	AT-skew	GC%	GC-skew	AT-skew
Sporophila bouvreuil	46.88	-0.384	0.120	48.06	-0.404	0.059	41.52	0.023	0.037	46.39	-0.119	0.223
Sporophila hypoxantha	46.93	-0.384	0.120	48.11	-0.404	0.058	41.41	0.023	0.038	46.22	-0.119	0.221
Sporophila iberaensis	47.08	-0.384	0.120	48.07	-0.405	0.061	41.37	0.045	0.027	44.97	-0.106	0.238
Sporophila maximiliani	46.91	-0.385	0.119	48.01	-0.407	0.058	42.41	0.024	0.048	46.23	-0.115	0.225
Sporophila melanogaster	46.92	-0.405	0.126	47.84	-0.407	0.061	41.26	0.028	0.029	54.84	-0.122	0.237
Sporophila minuta	46.96	-0.386	0.122	48.14	-0.402	0.057	41.83	0.020	0.043	46.75	-0.131	0.238
Sporophila nigricollis	46.66	-0.380	0.119	47.79	-0.399	0.060	42.09	0.021	0.044	46.14	-0.119	0.227
Sporophila nigrorufa	46.88	-0.405	0.127	47.84	-0.405	0.062	41.19	0.025	0.029	44.89	-0.105	0.238
Sporophila pileata	47.07	-0.406	0.125	48.07	-0.404	0.061	41.31	0.014	0.039	44.95	-0.106	0.237
Sporophila ruficollis	46.91	-0.384	0.120	48.09	-0.404	0.059	41.45	0.025	0.038	46.33	-0.120	0.222
**Average**	46.92	-0.390	0.122	48.00	-0.404	0.060	41.58	0.025	0.037	46.77	-0.116	0.231
**Standard deviation**	0.11	0.01	0.00	0.12	0.00	0.00	0.38	0.01	0.01	2.77	0.01	0.01
