# Supplementary material for: Mitochondrion genomes of seven species of the endangered genus Sporophila (Passeriformes: Thraupidae)
Source: Genet Mol Biol. 2024 Apr 5;47(1):e20230172. doi: 10.1590/1678-4685-GMB-2023-0172 (PMC10995768; doi:10.1590/1678-4685-GMB-2023-0172)
Supplement: Table S3 - [file 1415-4757-GMB-47-1-e20230172-s3.pdf]

Genetics and Molecular Biology
https://doi.org/10.1590/1678-4685-GMB-2023-0172

Supplementary Material to "Mitochondrion genomes of seven species of the endangered genus *Sporophila* (Passeriformes: Thraupidae)"

Table S3 - Number of codons and Relative Synonymous Codon Usage (RSCU) for ten *Sporophila* mitochondrial genomes. AA code: amino acid code.

Species	Codon	AA code	Count	RSCU
Sporophila bouvreuil	GCU	A	42	0.55
Sporophila bouvreuil	GCC	A	171	2.22
Sporophila bouvreuil	GCA	A	82	1.06
Sporophila bouvreuil	GCG	A	13	0.17
Sporophila bouvreuil	CGU	R	9	0.5
Sporophila bouvreuil	CGC	R	15	0.83
Sporophila bouvreuil	CGA	R	42	2.33
Sporophila bouvreuil	CGG	R	6	0.33
Sporophila bouvreuil	AAU	N	21	0.32
Sporophila bouvreuil	AAC	N	111	1.68
Sporophila bouvreuil	GAU	D	11	0.32

**Species**	**Codon**	**AA code**	**Count**	**RSCU**
Sporophila bouvreuil	GAC	D	58	1.68
Sporophila bouvreuil	UGU	C	4	0.27
Sporophila bouvreuil	UGC	C	26	1.73
Sporophila bouvreuil	CAA	Q	83	1.77
Sporophila bouvreuil	CAG	Q	11	0.23
Sporophila bouvreuil	GAA	E	76	1.71
Sporophila bouvreuil	GAG	E	13	0.29
Sporophila bouvreuil	GGU	G	25	0.45
Sporophila bouvreuil	GGC	G	71	1.27
Sporophila bouvreuil	GGA	G	101	1.8
Sporophila bouvreuil	GGG	G	27	0.48
Sporophila bouvreuil	CAU	H	17	0.34

Species	Codon	AA code	Count	RSCU
Sporophila bouvreuil	CAC	H	84	1.66
Sporophila bouvreuil	AUU	I	74	0.51
Sporophila bouvreuil	AUC	I	218	1.49
Sporophila bouvreuil	UUA	L	52	0.46
Sporophila bouvreuil	UUG	L	22	0.19
Sporophila bouvreuil	CUU	L	57	0.5
Sporophila bouvreuil	CUC	L	185	1.64
Sporophila bouvreuil	CUA	L	303	2.68
Sporophila bouvreuil	CUG	L	59	0.52
Sporophila bouvreuil	AAA	K	77	1.81
Sporophila bouvreuil	AAG	K	8	0.19
Sporophila bouvreuil	AUA	M	110	1.46

Species	Codon	AA code	Count	RSCU
Sporophila bouvreuil	AUG	M	41	0.54
Sporophila bouvreuil	UUU	F	32	0.29
Sporophila bouvreuil	UUC	F	188	1.71
Sporophila bouvreuil	CCU	P	37	0.65
Sporophila bouvreuil	CCC	P	102	1.81
Sporophila bouvreuil	CCA	P	80	1.42
Sporophila bouvreuil	CCG	P	7	0.12
Sporophila bouvreuil	UCU	S	31	0.65
Sporophila bouvreuil	UCC	S	99	2.08
Sporophila bouvreuil	UCA	S	91	1.91
Sporophila bouvreuil	UCG	S	4	0.08
Sporophila bouvreuil	AGU	S	5	0.1

Species	Codon	AA code	Count	RSCU
Sporophila bouvreuil	AGC	S	56	1.17
Sporophila bouvreuil	ACU	T	48	0.61
Sporophila bouvreuil	ACC	T	131	1.66
Sporophila bouvreuil	ACA	T	126	1.59
Sporophila bouvreuil	ACG	T	11	0.14
Sporophila bouvreuil	UGA	W	101	1.91
Sporophila bouvreuil	UGG	W	5	0.09
Sporophila bouvreuil	UAU	Y	22	0.39
Sporophila bouvreuil	UAC	Y	90	1.61
Sporophila bouvreuil	GUU	V	36	0.74
Sporophila bouvreuil	GUC	V	69	1.42
Sporophila bouvreuil	GUA	V	70	1.44

Species	Codon	AA code	Count	RSCU
Sporophila bouvreuil	GUG	V	20	0.41
Sporophila bouvreuil	UAA	*	6	2.18
Sporophila bouvreuil	UAG	*	2	0.73
Sporophila bouvreuil	AGA	*	2	0.73
Sporophila bouvreuil	AGG	*	1	0.36
Sporophila hypoxantha	GCU	A	42	0.55
Sporophila hypoxantha	GCC	A	170	2.21
Sporophila hypoxantha	GCA	A	82	1.06
Sporophila hypoxantha	GCG	A	14	0.18
Sporophila hypoxantha	CGU	R	8	0.44
Sporophila hypoxantha	CGC	R	16	0.89
Sporophila hypoxantha	CGA	R	42	2.33

Species	Codon	AA code	Count	RSCU
Sporophila hypoxantha	CGG	R	6	0.33
Sporophila hypoxantha	AAU	N	20	0.31
Sporophila hypoxantha	AAC	N	111	1.69
Sporophila hypoxantha	GAU	D	9	0.26
Sporophila hypoxantha	GAC	D	60	1.74
Sporophila hypoxantha	UGU	C	4	0.27
Sporophila hypoxantha	UGC	C	26	1.73
Sporophila hypoxantha	CAA	Q	82	1.74
Sporophila hypoxantha	CAG	Q	12	0.26
Sporophila hypoxantha	GAA	E	75	1.69
Sporophila hypoxantha	GAG	E	14	0.31
Sporophila hypoxantha	GGU	G	22	0.39

Species	Codon	AA code	Count	RSCU
Sporophila hypoxantha	GGC	G	74	1.32
Sporophila hypoxantha	GGA	G	100	1.79
Sporophila hypoxantha	GGG	G	28	0.5
Sporophila hypoxantha	CAU	H	19	0.38
Sporophila hypoxantha	CAC	H	82	1.62
Sporophila hypoxantha	AUU	I	74	0.51
Sporophila hypoxantha	AUC	I	219	1.49
Sporophila hypoxantha	UUA	L	50	0.44
Sporophila hypoxantha	UUG	L	22	0.2
Sporophila hypoxantha	CUU	L	56	0.5
Sporophila hypoxantha	CUC	L	187	1.66
Sporophila hypoxantha	CUA	L	305	2.71

Species	Codon	AA code	Count	RSCU
Sporophila hypoxantha	CUG	L	56	0.5
Sporophila hypoxantha	AAA	K	76	1.79
Sporophila hypoxantha	AAG	K	9	0.21
Sporophila hypoxantha	AUA	M	111	1.48
Sporophila hypoxantha	AUG	M	39	0.52
Sporophila hypoxantha	UUU	F	36	0.33
Sporophila hypoxantha	UUC	F	184	1.67
Sporophila hypoxantha	CCU	P	40	0.7
Sporophila hypoxantha	CCC	P	100	1.76
Sporophila hypoxantha	CCA	P	80	1.41
Sporophila hypoxantha	CCG	P	7	0.12
Sporophila hypoxantha	UCU	S	33	0.69

Species	Codon	AA code	Count	RSCU
Sporophila hypoxantha	UCC	S	98	2.05
Sporophila hypoxantha	UCA	S	90	1.88
Sporophila hypoxantha	UCG	S	4	0.08
Sporophila hypoxantha	AGU	S	6	0.13
Sporophila hypoxantha	AGC	S	56	1.17
Sporophila hypoxantha	ACU	T	45	0.57
Sporophila hypoxantha	ACC	T	133	1.69
Sporophila hypoxantha	ACA	T	126	1.6
Sporophila hypoxantha	ACG	T	11	0.14
Sporophila hypoxantha	UGA	W	103	1.94
Sporophila hypoxantha	UGG	W	3	0.06
Sporophila hypoxantha	UAU	Y	23	0.41

Species	Codon	AA code	Count	RSCU
Sporophila hypoxantha	UAC	Y	89	1.59
Sporophila hypoxantha	GUU	V	35	0.71
Sporophila hypoxantha	GUC	V	70	1.41
Sporophila hypoxantha	GUA	V	71	1.43
Sporophila hypoxantha	GUG	V	22	0.44
Sporophila hypoxantha	UAA	*	5	2
Sporophila hypoxantha	UAG	*	2	0.8
Sporophila hypoxantha	AGA	*	2	0.8
Sporophila hypoxantha	AGG	*	1	0.4
Sporophila iberaensis	GCU	A	45	0.59
Sporophila iberaensis	GCC	A	167	2.18
Sporophila iberaensis	GCA	A	82	1.07

Species	Codon	AA code	Count	RSCU
Sporophila iberaensis	GCG	A	12	0.16
Sporophila iberaensis	CGU	R	10	0.56
Sporophila iberaensis	CGC	R	14	0.78
Sporophila iberaensis	CGA	R	44	2.44
Sporophila iberaensis	CGG	R	4	0.22
Sporophila iberaensis	AAU	N	19	0.29
Sporophila iberaensis	AAC	N	111	1.71
Sporophila iberaensis	GAU	D	8	0.23
Sporophila iberaensis	GAC	D	61	1.77
Sporophila iberaensis	UGU	C	4	0.26
Sporophila iberaensis	UGC	C	27	1.74
Sporophila iberaensis	CAA	Q	83	1.77

Species	Codon	AA code	Count	RSCU
Sporophila iberaensis	CAG	Q	11	0.23
Sporophila iberaensis	GAA	E	74	1.68
Sporophila iberaensis	GAG	E	14	0.32
Sporophila iberaensis	GGU	G	22	0.39
Sporophila iberaensis	GGC	G	74	1.32
Sporophila iberaensis	GGA	G	98	1.75
Sporophila iberaensis	GGG	G	30	0.54
Sporophila iberaensis	CAU	H	17	0.34
Sporophila iberaensis	CAC	H	84	1.66
Sporophila iberaensis	AUU	I	68	0.47
Sporophila iberaensis	AUC	I	223	1.53
Sporophila iberaensis	UUA	L	50	0.44

Species	Codon	AA code	Count	RSCU
Sporophila iberaensis	UUG	L	22	0.2
Sporophila iberaensis	CUU	L	57	0.51
Sporophila iberaensis	CUC	L	185	1.64
Sporophila iberaensis	CUA	L	308	2.74
Sporophila iberaensis	CUG	L	53	0.47
Sporophila iberaensis	AAA	K	76	1.79
Sporophila iberaensis	AAG	K	9	0.21
Sporophila iberaensis	AUA	M	112	1.49
Sporophila iberaensis	AUG	M	38	0.51
Sporophila iberaensis	UUU	F	33	0.3
Sporophila iberaensis	UUC	F	186	1.7
Sporophila iberaensis	CCU	P	37	0.65

Species	Codon	AA code	Count	RSCU
Sporophila iberaensis	CCC	P	103	1.81
Sporophila iberaensis	CCA	P	79	1.39
Sporophila iberaensis	CCG	P	8	0.14
Sporophila iberaensis	UCU	S	33	0.69
Sporophila iberaensis	UCC	S	97	2.03
Sporophila iberaensis	UCA	S	91	1.9
Sporophila iberaensis	UCG	S	4	0.08
Sporophila iberaensis	AGU	S	6	0.13
Sporophila iberaensis	AGC	S	56	1.17
Sporophila iberaensis	ACU	T	47	0.59
Sporophila iberaensis	ACC	T	131	1.66
Sporophila iberaensis	ACA	T	128	1.62

Species	Codon	AA code	Count	RSCU
Sporophila iberaensis	ACG	T	10	0.13
Sporophila iberaensis	UGA	W	103	1.94
Sporophila iberaensis	UGG	W	3	0.06
Sporophila iberaensis	UAU	Y	23	0.41
Sporophila iberaensis	UAC	Y	89	1.59
Sporophila iberaensis	GUU	V	37	0.75
Sporophila iberaensis	GUC	V	68	1.38
Sporophila iberaensis	GUA	V	70	1.42
Sporophila iberaensis	GUG	V	22	0.45
Sporophila iberaensis	UAA	*	6	2.18
Sporophila iberaensis	UAG	*	2	0.73
Sporophila iberaensis	AGA	*	2	0.73

Species	Codon	AA code	Count	RSCU
Sporophila iberaensis	AGG	*	1	0.36
Sporophila maximiliani	GCU	A	44	0.57
Sporophila maximiliani	GCC	A	169	2.19
Sporophila maximiliani	GCA	A	84	1.09
Sporophila maximiliani	GCG	A	12	0.16
Sporophila maximiliani	CGU	R	4	0.22
Sporophila maximiliani	CGC	R	20	1.11
Sporophila maximiliani	CGA	R	42	2.33
Sporophila maximiliani	CGG	R	6	0.33
Sporophila maximiliani	AAU	N	19	0.29
Sporophila maximiliani	AAC	N	113	1.71
Sporophila maximiliani	GAU	D	11	0.35

Species	Codon	AA code	Count	RSCU
Sporophila maximiliani	GAC	D	52	1.65
Sporophila maximiliani	UGU	C	6	0.39
Sporophila maximiliani	UGC	C	25	1.61
Sporophila maximiliani	CAA	Q	83	1.77
Sporophila maximiliani	CAG	Q	11	0.23
Sporophila maximiliani	GAA	E	74	1.64
Sporophila maximiliani	GAG	E	16	0.36
Sporophila maximiliani	GGU	G	27	0.48
Sporophila maximiliani	GGC	G	74	1.32
Sporophila maximiliani	GGA	G	94	1.67
Sporophila maximiliani	GGG	G	30	0.53
Sporophila maximiliani	CAU	H	12	0.24

Species	Codon	AA code	Count	RSCU
Sporophila maximiliani	CAC	H	90	1.76
Sporophila maximiliani	AUU	I	77	0.54
Sporophila maximiliani	AUC	I	208	1.46
Sporophila maximiliani	UUA	L	57	0.5
Sporophila maximiliani	UUG	L	20	0.18
Sporophila maximiliani	CUU	L	51	0.45
Sporophila maximiliani	CUC	L	195	1.73
Sporophila maximiliani	CUA	L	311	2.75
Sporophila maximiliani	CUG	L	44	0.39
Sporophila maximiliani	AAA	K	82	1.95
Sporophila maximiliani	AAG	K	2	0.05
Sporophila maximiliani	AUA	M	110	1.45

Species	Codon	AA code	Count	RSCU
Sporophila maximiliani	AUG	M	42	0.55
Sporophila maximiliani	UUU	F	33	0.3
Sporophila maximiliani	UUC	F	190	1.7
Sporophila maximiliani	CCU	P	30	0.54
Sporophila maximiliani	CCC	P	107	1.91
Sporophila maximiliani	CCA	P	75	1.34
Sporophila maximiliani	CCG	P	12	0.21
Sporophila maximiliani	UCU	S	35	0.72
Sporophila maximiliani	UCC	S	94	1.94
Sporophila maximiliani	UCA	S	85	1.76
Sporophila maximiliani	UCG	S	10	0.21
Sporophila maximiliani	AGU	S	8	0.17

Species	Codon	AA code	Count	RSCU
Sporophila maximiliani	AGC	S	58	1.2
Sporophila maximiliani	ACU	T	58	0.73
Sporophila maximiliani	ACC	T	123	1.54
Sporophila maximiliani	ACA	T	133	1.67
Sporophila maximiliani	ACG	T	5	0.06
Sporophila maximiliani	UGA	W	102	1.94
Sporophila maximiliani	UGG	W	3	0.06
Sporophila maximiliani	UAU	Y	18	0.32
Sporophila maximiliani	UAC	Y	93	1.68
Sporophila maximiliani	GUU	V	40	0.81
Sporophila maximiliani	GUC	V	69	1.39
Sporophila maximiliani	GUA	V	70	1.41

Species	Codon	AA code	Count	RSCU
Sporophila maximiliani	GUG	V	19	0.38
Sporophila maximiliani	UAA	*	5	2
Sporophila maximiliani	UAG	*	2	0.8
Sporophila maximiliani	AGA	*	2	0.8
Sporophila maximiliani	AGG	*	1	0.4
Sporophila melanogaster	GCU	A	43	0.57
Sporophila melanogaster	GCC	A	166	2.18
Sporophila melanogaster	GCA	A	82	1.08
Sporophila melanogaster	GCG	A	13	0.17
Sporophila melanogaster	CGU	R	9	0.51

Species	Codon	AA code	Count	RSCU
Sporophila melanogaster	CGC	R	13	0.74
Sporophila melanogaster	CGA	R	43	2.46
Sporophila melanogaster	CGG	R	5	0.29
Sporophila melanogaster	AAU	N	19	0.29
Sporophila melanogaster	AAC	N	112	1.71
Sporophila melanogaster	GAU	D	9	0.26
Sporophila melanogaster	GAC	D	60	1.74
Sporophila melanogaster	UGU	C	4	0.28

Species	Codon	AA code	Count	RSCU
Sporophila melanogaster	UGC	C	25	1.72
Sporophila melanogaster	CAA	Q	81	1.74
Sporophila melanogaster	CAG	Q	12	0.26
Sporophila melanogaster	GAA	E	75	1.69
Sporophila melanogaster	GAG	E	14	0.31
Sporophila melanogaster	GGU	G	23	0.42
Sporophila melanogaster	GGC	G	73	1.33
Sporophila melanogaster	GGA	G	97	1.77

Species	Codon	AA code	Count	RSCU
Sporophila melanogaster	GGG	G	26	0.47
Sporophila melanogaster	CAU	H	17	0.34
Sporophila melanogaster	CAC	H	82	1.66
Sporophila melanogaster	AUU	I	71	0.49
Sporophila melanogaster	AUC	I	220	1.51
Sporophila melanogaster	UUA	L	49	0.44
Sporophila melanogaster	UUG	L	20	0.18
Sporophila melanogaster	CUU	L	54	0.49

Species	Codon	AA code	Count	RSCU
Sporophila melanogaster	CUC	L	185	1.67
Sporophila melanogaster	CUA	L	309	2.78
Sporophila melanogaster	CUG	L	49	0.44
Sporophila melanogaster	AAA	K	76	1.81
Sporophila melanogaster	AAG	K	8	0.19
Sporophila melanogaster	AUA	M	110	1.48
Sporophila melanogaster	AUG	M	39	0.52
Sporophila melanogaster	UUU	F	34	0.31
Sporophila melanogaster	UUC	F	186	1.69

Species	Codon	AA code	Count	RSCU
Sporophila melanogaster	CCU	P	36	0.65
Sporophila melanogaster	CCC	P	101	1.83
Sporophila melanogaster	CCA	P	77	1.39
Sporophila melanogaster	CCG	P	7	0.13
Sporophila melanogaster	UCU	S	32	0.67
Sporophila melanogaster	UCC	S	98	2.06
Sporophila melanogaster	UCA	S	91	1.91
Sporophila melanogaster	UCG	S	4	0.08

Species	Codon	AA code	Count	RSCU
Sporophila melanogaster	AGU	S	6	0.13
Sporophila melanogaster	AGC	S	55	1.15
Sporophila melanogaster	ACU	T	47	0.6
Sporophila melanogaster	ACC	T	129	1.64
Sporophila melanogaster	ACA	T	128	1.63
Sporophila melanogaster	ACG	T	10	0.13
Sporophila melanogaster	UGA	W	103	1.94
Sporophila melanogaster	UGG	W	3	0.06

Species	Codon	AA code	Count	RSCU
Sporophila melanogaster	UAU	Y	21	0.38
Sporophila melanogaster	UAC	Y	91	1.63
Sporophila melanogaster	GUU	V	38	0.78
Sporophila melanogaster	GUC	V	66	1.35
Sporophila melanogaster	GUA	V	70	1.44
Sporophila melanogaster	GUG	V	21	0.43
Sporophila melanogaster	UAA	*	6	2.18
Sporophila melanogaster	UAG	*	2	0.73

Species	Codon	AA code	Count	RSCU
Sporophila melanogaster	AGA	*	2	0.73
Sporophila melanogaster	AGG	*	1	0.36
Sporophila minuta	GCU	A	50	0.66
Sporophila minuta	GCC	A	161	2.11
Sporophila minuta	GCA	A	82	1.08
Sporophila minuta	GCG	A	12	0.16
Sporophila minuta	CGU	R	9	0.5
Sporophila minuta	CGC	R	16	0.89
Sporophila minuta	CGA	R	39	2.17
Sporophila minuta	CGG	R	8	0.44
Sporophila minuta	AAU	N	21	0.31

Species	Codon	AA code	Count	RSCU
Sporophila minuta	AAC	N	115	1.69
Sporophila minuta	GAU	D	11	0.33
Sporophila minuta	GAC	D	56	1.67
Sporophila minuta	UGU	C	7	0.47
Sporophila minuta	UGC	C	23	1.53
Sporophila minuta	CAA	Q	86	1.83
Sporophila minuta	CAG	Q	8	0.17
Sporophila minuta	GAA	E	75	1.7
Sporophila minuta	GAG	E	13	0.3
Sporophila minuta	GGU	G	22	0.39
Sporophila minuta	GGC	G	73	1.31
Sporophila minuta	GGA	G	94	1.69

Species	Codon	AA code	Count	RSCU
Sporophila minuta	GGG	G	34	0.61
Sporophila minuta	CAU	H	16	0.31
Sporophila minuta	CAC	H	87	1.69
Sporophila minuta	AUU	I	72	0.49
Sporophila minuta	AUC	I	224	1.51
Sporophila minuta	UUA	L	42	0.37
Sporophila minuta	UUG	L	26	0.23
Sporophila minuta	CUU	L	59	0.52
Sporophila minuta	CUC	L	182	1.62
Sporophila minuta	CUA	L	308	2.73
Sporophila minuta	CUG	L	59	0.52
Sporophila minuta	AAA	K	77	1.81

Species	Codon	AA code	Count	RSCU
Sporophila minuta	AAG	K	8	0.19
Sporophila minuta	AUA	M	107	1.49
Sporophila minuta	AUG	M	37	0.51
Sporophila minuta	UUU	F	38	0.35
Sporophila minuta	UUC	F	179	1.65
Sporophila minuta	CCU	P	38	0.66
Sporophila minuta	CCC	P	100	1.75
Sporophila minuta	CCA	P	84	1.47
Sporophila minuta	CCG	P	7	0.12
Sporophila minuta	UCU	S	38	0.81
Sporophila minuta	UCC	S	97	2.06
Sporophila minuta	UCA	S	77	1.64

Species	Codon	AA code	Count	RSCU
Sporophila minuta	UCG	S	10	0.21
Sporophila minuta	AGU	S	10	0.21
Sporophila minuta	AGC	S	50	1.06
Sporophila minuta	ACU	T	37	0.46
Sporophila minuta	ACC	T	141	1.75
Sporophila minuta	ACA	T	131	1.62
Sporophila minuta	ACG	T	14	0.17
Sporophila minuta	UGA	W	100	1.89
Sporophila minuta	UGG	W	6	0.11
Sporophila minuta	UAU	Y	21	0.38
Sporophila minuta	UAC	Y	91	1.63
Sporophila minuta	GUU	V	40	0.8

Species	Codon	AA code	Count	RSCU
Sporophila minuta	GUC	V	65	1.31
Sporophila minuta	GUA	V	72	1.45
Sporophila minuta	GUG	V	22	0.44
Sporophila minuta	UAA	*	8	2.91
Sporophila minuta	UAG	*	0	0
Sporophila minuta	AGA	*	2	0.73
Sporophila minuta	AGG	*	1	0.36
Sporophila nigricollis	GCU	A	50	0.64
Sporophila nigricollis	GCC	A	160	2.04
Sporophila nigricollis	GCA	A	88	1.12
Sporophila nigricollis	GCG	A	15	0.19
Sporophila nigricollis	CGU	R	12	0.66

Species	Codon	AA code	Count	RSCU
Sporophila nigricollis	CGC	R	11	0.6
Sporophila nigricollis	CGA	R	40	2.19
Sporophila nigricollis	CGG	R	10	0.55
Sporophila nigricollis	AAU	N	21	0.31
Sporophila nigricollis	AAC	N	113	1.69
Sporophila nigricollis	GAU	D	13	0.4
Sporophila nigricollis	GAC	D	52	1.6
Sporophila nigricollis	UGU	C	5	0.33
Sporophila nigricollis	UGC	C	25	1.67
Sporophila nigricollis	CAA	Q	82	1.76
Sporophila nigricollis	CAG	Q	11	0.24
Sporophila nigricollis	GAA	E	79	1.76

Species	Codon	AA code	Count	RSCU
Sporophila nigricollis	GAG	E	11	0.24
Sporophila nigricollis	GGU	G	22	0.39
Sporophila nigricollis	GGC	G	74	1.32
Sporophila nigricollis	GGA	G	96	1.71
Sporophila nigricollis	GGG	G	33	0.59
Sporophila nigricollis	CAU	H	19	0.36
Sporophila nigricollis	CAC	H	86	1.64
Sporophila nigricollis	AUU	I	77	0.53
Sporophila nigricollis	AUC	I	211	1.47
Sporophila nigricollis	UUA	L	54	0.48
Sporophila nigricollis	UUG	L	18	0.16
Sporophila nigricollis	CUU	L	53	0.47

Species	Codon	AA code	Count	RSCU
Sporophila nigricollis	CUC	L	183	1.62
Sporophila nigricollis	CUA	L	319	2.83
Sporophila nigricollis	CUG	L	50	0.44
Sporophila nigricollis	AAA	K	80	1.88
Sporophila nigricollis	AAG	K	5	0.12
Sporophila nigricollis	AUA	M	105	1.43
Sporophila nigricollis	AUG	M	42	0.57
Sporophila nigricollis	UUU	F	42	0.38
Sporophila nigricollis	UUC	F	177	1.62
Sporophila nigricollis	CCU	P	33	0.59
Sporophila nigricollis	CCC	P	97	1.72
Sporophila nigricollis	CCA	P	86	1.53

Species	Codon	AA code	Count	RSCU
Sporophila nigricollis	CCG	P	9	0.16
Sporophila nigricollis	UCU	S	32	0.67
Sporophila nigricollis	UCC	S	95	2
Sporophila nigricollis	UCA	S	89	1.87
Sporophila nigricollis	UCG	S	8	0.17
Sporophila nigricollis	AGU	S	7	0.15
Sporophila nigricollis	AGC	S	54	1.14
Sporophila nigricollis	ACU	T	45	0.57
Sporophila nigricollis	ACC	T	136	1.72
Sporophila nigricollis	ACA	T	126	1.59
Sporophila nigricollis	ACG	T	9	0.11
Sporophila nigricollis	UGA	W	101	1.91

Species	Codon	AA code	Count	RSCU
Sporophila nigricollis	UGG	W	5	0.09
Sporophila nigricollis	UAU	Y	18	0.32
Sporophila nigricollis	UAC	Y	94	1.68
Sporophila nigricollis	GUU	V	43	0.86
Sporophila nigricollis	GUC	V	65	1.31
Sporophila nigricollis	GUA	V	75	1.51
Sporophila nigricollis	GUG	V	16	0.32
Sporophila nigricollis	UAA	*	6	2.4
Sporophila nigricollis	UAG	*	1	0.4
Sporophila nigricollis	AGA	*	2	0.8
Sporophila nigricollis	AGG	*	1	0.4
Sporophila nigrorufa	GCU	A	41	0.54

Species	Codon	AA code	Count	RSCU
Sporophila nigrorufa	GCC	A	166	2.21
Sporophila nigrorufa	GCA	A	81	1.08
Sporophila nigrorufa	GCG	A	13	0.17
Sporophila nigrorufa	CGU	R	8	0.45
Sporophila nigrorufa	CGC	R	15	0.85
Sporophila nigrorufa	CGA	R	43	2.42
Sporophila nigrorufa	CGG	R	5	0.28
Sporophila nigrorufa	AAU	N	17	0.26
Sporophila nigrorufa	AAC	N	112	1.74
Sporophila nigrorufa	GAU	D	9	0.26
Sporophila nigrorufa	GAC	D	59	1.74
Sporophila nigrorufa	UGU	C	4	0.27

Species	Codon	AA code	Count	RSCU
Sporophila nigrorufa	UGC	C	26	1.73
Sporophila nigrorufa	CAA	Q	80	1.74
Sporophila nigrorufa	CAG	Q	12	0.26
Sporophila nigrorufa	GAA	E	75	1.69
Sporophila nigrorufa	GAG	E	14	0.31
Sporophila nigrorufa	GGU	G	23	0.42
Sporophila nigrorufa	GGC	G	72	1.31
Sporophila nigrorufa	GGA	G	98	1.78
Sporophila nigrorufa	GGG	G	27	0.49
Sporophila nigrorufa	CAU	H	18	0.37
Sporophila nigrorufa	CAC	H	79	1.63
Sporophila nigrorufa	AUU	I	71	0.49

Species	Codon	AA code	Count	RSCU
Sporophila nigrorufa	AUC	I	219	1.51
Sporophila nigrorufa	UUA	L	47	0.43
Sporophila nigrorufa	UUG	L	23	0.21
Sporophila nigrorufa	CUU	L	52	0.47
Sporophila nigrorufa	CUC	L	184	1.67
Sporophila nigrorufa	CUA	L	305	2.76
Sporophila nigrorufa	CUG	L	51	0.46
Sporophila nigrorufa	AAA	K	77	1.81
Sporophila nigrorufa	AAG	K	8	0.19
Sporophila nigrorufa	AUA	M	109	1.47
Sporophila nigrorufa	AUG	M	39	0.53
Sporophila nigrorufa	UUU	F	34	0.31

Species	Codon	AA code	Count	RSCU
Sporophila nigrorufa	UUC	F	185	1.69
Sporophila nigrorufa	CCU	P	37	0.66
Sporophila nigrorufa	CCC	P	100	1.79
Sporophila nigrorufa	CCA	P	79	1.41
Sporophila nigrorufa	CCG	P	8	0.14
Sporophila nigrorufa	UCU	S	32	0.68
Sporophila nigrorufa	UCC	S	96	2.03
Sporophila nigrorufa	UCA	S	91	1.92
Sporophila nigrorufa	UCG	S	4	0.08
Sporophila nigrorufa	AGU	S	6	0.13
Sporophila nigrorufa	AGC	S	55	1.16
Sporophila nigrorufa	ACU	T	44	0.57

Species	Codon	AA code	Count	RSCU
Sporophila nigrorufa	ACC	T	129	1.67
Sporophila nigrorufa	ACA	T	126	1.63
Sporophila nigrorufa	ACG	T	10	0.13
Sporophila nigrorufa	UGA	W	103	1.94
Sporophila nigrorufa	UGG	W	3	0.06
Sporophila nigrorufa	UAU	Y	19	0.35
Sporophila nigrorufa	UAC	Y	90	1.65
Sporophila nigrorufa	GUU	V	38	0.79
Sporophila nigrorufa	GUC	V	63	1.31
Sporophila nigrorufa	GUA	V	70	1.46
Sporophila nigrorufa	GUG	V	21	0.44
Sporophila nigrorufa	UAA	*	6	2.18

Species	Codon	AA code	Count	RSCU
Sporophila nigrorufa	UAG	*	2	0.73
Sporophila nigrorufa	AGA	*	2	0.73
Sporophila nigrorufa	AGG	*	1	0.36
Sporophila pileata	GCU	A	42	0.55
Sporophila pileata	GCC	A	169	2.2
Sporophila pileata	GCA	A	82	1.07
Sporophila pileata	GCG	A	14	0.18
Sporophila pileata	CGU	R	7	0.39
Sporophila pileata	CGC	R	16	0.9
Sporophila pileata	CGA	R	42	2.37
Sporophila pileata	CGG	R	6	0.34
Sporophila pileata	AAU	N	19	0.29

Species	Codon	AA code	Count	RSCU
Sporophila pileata	AAC	N	111	1.71
Sporophila pileata	GAU	D	10	0.29
Sporophila pileata	GAC	D	58	1.71
Sporophila pileata	UGU	C	4	0.27
Sporophila pileata	UGC	C	26	1.73
Sporophila pileata	CAA	Q	81	1.76
Sporophila pileata	CAG	Q	11	0.24
Sporophila pileata	GAA	E	75	1.7
Sporophila pileata	GAG	E	13	0.3
Sporophila pileata	GGU	G	21	0.37
Sporophila pileata	GGC	G	74	1.31
Sporophila pileata	GGA	G	100	1.77

Species	Codon	AA code	Count	RSCU
Sporophila pileata	GGG	G	31	0.55
Sporophila pileata	CAU	H	19	0.38
Sporophila pileata	CAC	H	81	1.62
Sporophila pileata	AUU	I	73	0.5
Sporophila pileata	AUC	I	217	1.5
Sporophila pileata	UUA	L	48	0.43
Sporophila pileata	UUG	L	21	0.19
Sporophila pileata	CUU	L	55	0.5
Sporophila pileata	CUC	L	187	1.68
Sporophila pileata	CUA	L	303	2.73
Sporophila pileata	CUG	L	52	0.47
Sporophila pileata	AAA	K	77	1.81

Species	Codon	AA code	Count	RSCU
Sporophila pileata	AAG	K	8	0.19
Sporophila pileata	AUA	M	107	1.47
Sporophila pileata	AUG	M	39	0.53
Sporophila pileata	UUU	F	35	0.32
Sporophila pileata	UUC	F	183	1.68
Sporophila pileata	CCU	P	34	0.61
Sporophila pileata	CCC	P	102	1.83
Sporophila pileata	CCA	P	80	1.43
Sporophila pileata	CCG	P	7	0.13
Sporophila pileata	UCU	S	32	0.67
Sporophila pileata	UCC	S	99	2.08
Sporophila pileata	UCA	S	90	1.89

Species	Codon	AA code	Count	RSCU
Sporophila pileata	UCG	S	4	0.08
Sporophila pileata	AGU	S	5	0.1
Sporophila pileata	AGC	S	56	1.17
Sporophila pileata	ACU	T	44	0.56
Sporophila pileata	ACC	T	133	1.69
Sporophila pileata	ACA	T	128	1.63
Sporophila pileata	ACG	T	10	0.13
Sporophila pileata	UGA	W	101	1.94
Sporophila pileata	UGG	W	3	0.06
Sporophila pileata	UAU	Y	22	0.4
Sporophila pileata	UAC	Y	89	1.6
Sporophila pileata	GUU	V	35	0.72

Species	Codon	AA code	Count	RSCU
Sporophila pileata	GUC	V	68	1.39
Sporophila pileata	GUA	V	72	1.48
Sporophila pileata	GUG	V	20	0.41
Sporophila pileata	UAA	*	5	2
Sporophila pileata	UAG	*	2	0.8
Sporophila pileata	AGA	*	2	0.8
Sporophila pileata	AGG	*	1	0.4
Sporophila ruficollis	GCU	A	42	0.55
Sporophila ruficollis	GCC	A	170	2.21
Sporophila ruficollis	GCA	A	82	1.06
Sporophila ruficollis	GCG	A	14	0.18
Sporophila ruficollis	CGU	R	8	0.44

Species	Codon	AA code	Count	RSCU
Sporophila ruficollis	CGC	R	16	0.89
Sporophila ruficollis	CGA	R	42	2.33
Sporophila ruficollis	CGG	R	6	0.33
Sporophila ruficollis	AAU	N	20	0.31
Sporophila ruficollis	AAC	N	111	1.69
Sporophila ruficollis	GAU	D	9	0.26
Sporophila ruficollis	GAC	D	60	1.74
Sporophila ruficollis	UGU	C	4	0.27
Sporophila ruficollis	UGC	C	26	1.73
Sporophila ruficollis	CAA	Q	82	1.74
Sporophila ruficollis	CAG	Q	12	0.26
Sporophila ruficollis	GAA	E	75	1.69

Species	Codon	AA code	Count	RSCU
Sporophila ruficollis	GAG	E	14	0.31
Sporophila ruficollis	GGU	G	21	0.38
Sporophila ruficollis	GGC	G	75	1.34
Sporophila ruficollis	GGA	G	100	1.79
Sporophila ruficollis	GGG	G	28	0.5
Sporophila ruficollis	CAU	H	19	0.38
Sporophila ruficollis	CAC	H	82	1.62
Sporophila ruficollis	AUU	I	75	0.51
Sporophila ruficollis	AUC	I	219	1.49
Sporophila ruficollis	UUA	L	50	0.44
Sporophila ruficollis	UUG	L	22	0.2
Sporophila ruficollis	CUU	L	56	0.5

Species	Codon	AA code	Count	RSCU
Sporophila ruficollis	CUC	L	187	1.66
Sporophila ruficollis	CUA	L	305	2.71
Sporophila ruficollis	CUG	L	56	0.5
Sporophila ruficollis	AAA	K	76	1.79
Sporophila ruficollis	AAG	K	9	0.21
Sporophila ruficollis	AUA	M	111	1.48
Sporophila ruficollis	AUG	M	39	0.52
Sporophila ruficollis	UUU	F	36	0.33
Sporophila ruficollis	UUC	F	184	1.67
Sporophila ruficollis	CCU	P	40	0.7
Sporophila ruficollis	CCC	P	100	1.76
Sporophila ruficollis	CCA	P	80	1.41

Species	Codon	AA code	Count	RSCU
Sporophila ruficollis	CCG	P	7	0.12
Sporophila ruficollis	UCU	S	33	0.69
Sporophila ruficollis	UCC	S	98	2.05
Sporophila ruficollis	UCA	S	90	1.88
Sporophila ruficollis	UCG	S	4	0.08
Sporophila ruficollis	AGU	S	6	0.13
Sporophila ruficollis	AGC	S	56	1.17
Sporophila ruficollis	ACU	T	44	0.56
Sporophila ruficollis	ACC	T	133	1.69
Sporophila ruficollis	ACA	T	127	1.61
Sporophila ruficollis	ACG	T	11	0.14
Sporophila ruficollis	UGA	W	103	1.94

Species	Codon	AA code	Count	RSCU
Sporophila ruficollis	UGG	W	3	0.06
Sporophila ruficollis	UAU	Y	23	0.41
Sporophila ruficollis	UAC	Y	89	1.59
Sporophila ruficollis	GUU	V	36	0.73
Sporophila ruficollis	GUC	V	68	1.38
Sporophila ruficollis	GUA	V	71	1.44
Sporophila ruficollis	GUG	V	22	0.45
Sporophila ruficollis	UAA	*	6	2.18
Sporophila ruficollis	UAG	*	2	0.73
Sporophila ruficollis	AGA	*	2	0.73
Sporophila ruficollis	AGG	*	1	0.36
