# Supplementary material for: Mitochondrion genomes of seven species of the endangered genus Sporophila (Passeriformes: Thraupidae)
Source: Genet Mol Biol. 2024 Apr 5;47(1):e20230172. doi: 10.1590/1678-4685-GMB-2023-0172 (PMC10995768; doi:10.1590/1678-4685-GMB-2023-0172)
Supplement: Table S4 - [file 1415-4757-GMB-47-1-e20230172-s4.pdf]

Genetics and Molecular Biology
https://doi.org/10.1590/1678-4685-GMB-2023-0172

Supplementary Material to "Mitochondrion genomes of seven species of the endangered genus *Sporophila* (Passeriformes: Thraupidae)"

Table S4 - Nucleotide diversity of *Sporophila* species mitochondrial genomes. Midpoints are positions in base pairs (bp) along the mitogenomes. Pi values represent nucleotide diversity values.

Midpoint	Pi
1287	0.0103
1488	0.0101
1692	0.0127
1894	0.0104
2094	0.0130
2294	0.0122
2495	0.0130
2695	0.0210
2895	0.0351
3097	0.0467
3299	0.0533

**Midpoint**	**Pi**
3500	0.0479
3702	0.0402
3905	0.0312
4106	0.0388
4313	0.0510
4520	0.0543
4722	0.0472
4927	0.0343
5131	0.0231
5331	0.0205
5531	0.0266
5733	0.0402
5938	0.0400
6142	0.0427
6342	0.0417
6544	0.0412

Midpoint	Pi
6747	0.0352
6948	0.0263
7150	0.0322
7350	0.0379
7551	0.0431
7752	0.0407
7953	0.0399
8155	0.0420
8355	0.0409
8558	0.0425
8759	0.0383
8964	0.0357
9166	0.0309
9368	0.0300
9569	0.0382
9770	0.0371
9971	0.0373
10171	0.0365

Midpoint	Pi
10375	0.0441
10576	0.0477
10783	0.0425
10986	0.0401
11187	0.0388
11387	0.0385
11588	0.0299
11790	0.0330
11993	0.0348
12194	0.0427
12395	0.0413
12600	0.0432
12806	0.0424
13010	0.0434
13210	0.0459
13413	0.0482
13614	0.0428
13814	0.0373

Midpoint	Pi
14014	0.0338
14218	0.0346
14419	0.0324
14622	0.0324
14825	0.0350
15039	0.0419
15227	0.0452
**Average**	0.0359
**Median**	0.0384
**Maximum**	0.05426
**Minimum**	0.01007
