# Supplementary material for: Mitochondrion genomes of seven species of the endangered genus Sporophila (Passeriformes: Thraupidae)
Source: Genet Mol Biol. 2024 Apr 5;47(1):e20230172. doi: 10.1590/1678-4685-GMB-2023-0172 (PMC10995768; doi:10.1590/1678-4685-GMB-2023-0172)
Supplement: Figure S1 - [file 1415-4757-GMB-47-1-e20230172-s5.pdf]

Genetics and Molecular Biology
https://doi.org/10.1590/1678-4685-GMB-2023-0172

Supplementary Material to "Mitochondrion genomes of seven species of the endangered genus *Sporophila* (Passeriformes: Thraupidae)"

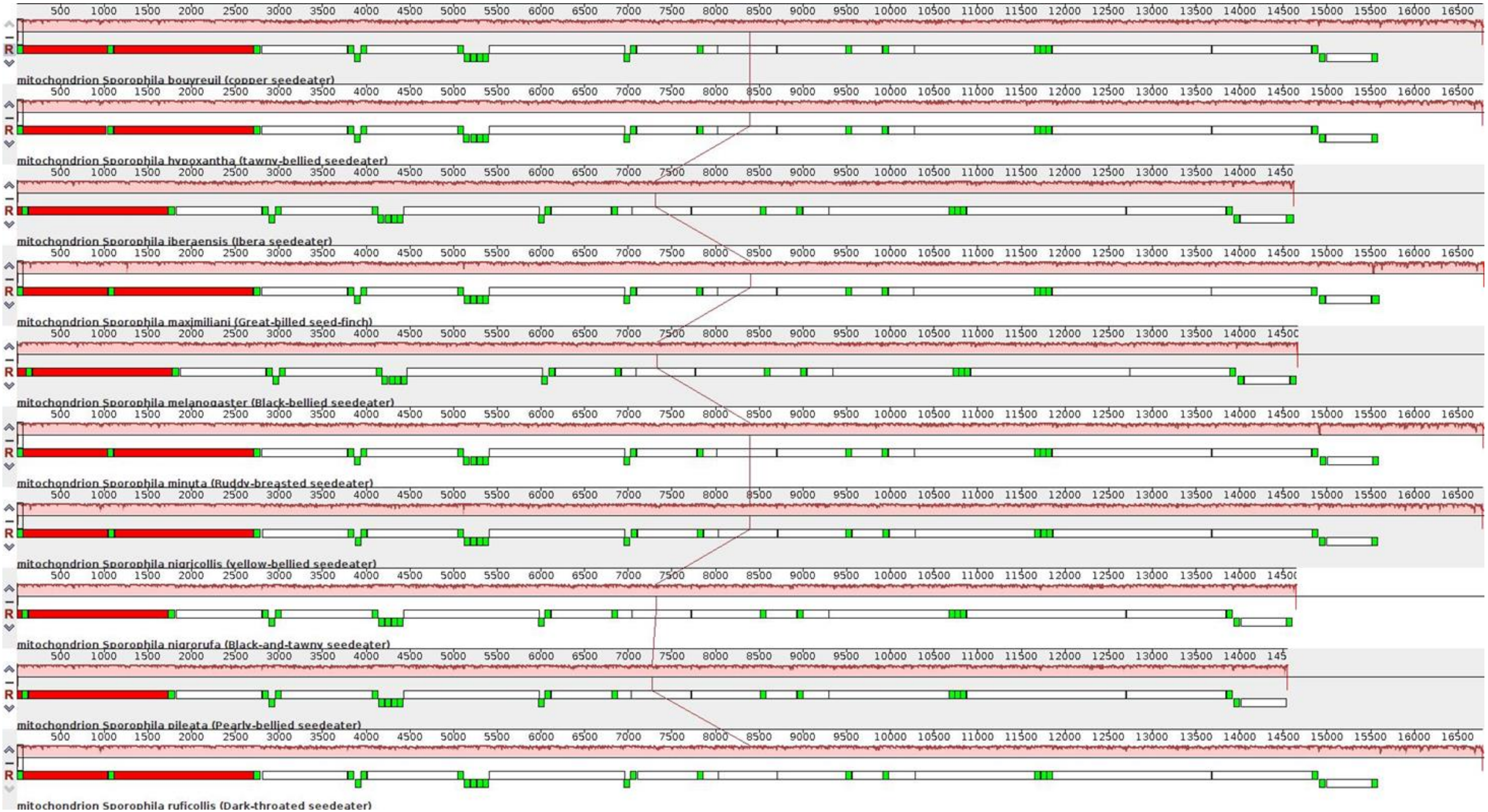

Figure S1 - Progressive alignment performed on MAUVE showing a unique similarity block between *Sporophila* species shown in the respective order: *S. bouvreuil*, *S. hypoxantha*, *S. iberaensis*, *S. maximiliani*, *S. melanogaster*, *S. minuta*, *S. nigricolli*, *S. nigrorufa*, *S. pileata*, *S. ruficollis*.
