# Supplementary material for: Mitochondrion genomes of seven species of the endangered genus Sporophila (Passeriformes: Thraupidae)
Source: Genet Mol Biol. 2024 Apr 5;47(1):e20230172. doi: 10.1590/1678-4685-GMB-2023-0172 (PMC10995768; doi:10.1590/1678-4685-GMB-2023-0172)
Supplement: Figure S2 - [file 1415-4757-GMB-47-1-e20230172-s6.pdf]

Genetics and Molecular Biology
https://doi.org/10.1590/1678-4685-GMB-2023-0172

Supplementary Material to "Mitochondrion genomes of seven species of the endangered genus *Sporophila* (Passeriformes: Thraupidae)"

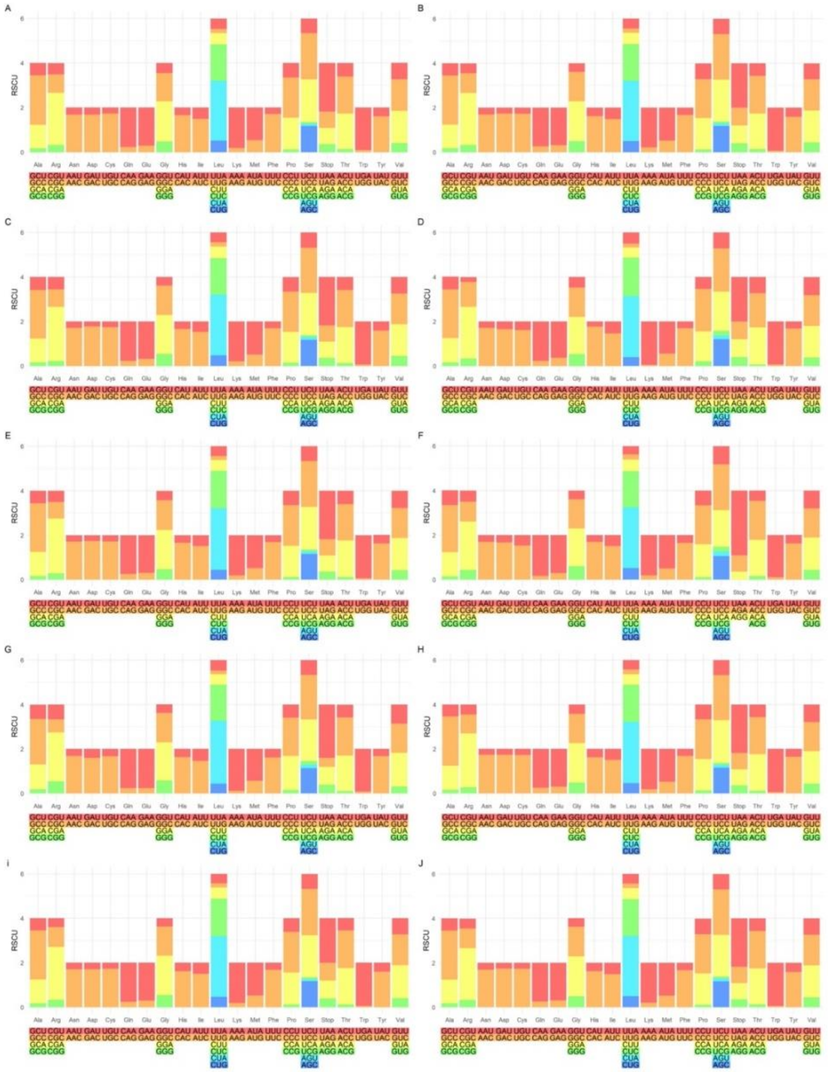

Figure S2 - Relative Synonymous Codon Usage (RSCU) analysis for all nine *Sporophila* species with complete mitochondrial genome currently available. A) *S. bouvreuil*; B) *S. hypoxantha*; C) *S. iberaensis*; D) *S. maximiliani*; E) *S. melanogaster*; F) *S. minuta*; G) *S. nigricollis*; H) *S. nigrorufa*; I) *S. pileata*; J) *S. ruficollis*.
