# Supplementary material for: Mitochondrion genomes of seven species of the endangered genus Sporophila (Passeriformes: Thraupidae)
Source: Genet Mol Biol. 2024 Apr 5;47(1):e20230172. doi: 10.1590/1678-4685-GMB-2023-0172 (PMC10995768; doi:10.1590/1678-4685-GMB-2023-0172)
Supplement: Figure S5 - [file 1415-4757-GMB-47-1-e20230172-s9.pdf]

*Genetics and Molecular Biology*
https://doi.org/10.1590/1678-4685-GMB-2023-0172

# Supplementary Material to "Mitochondrion genomes of seven species of the endangered genus *Sporophila* (Passeriformes: Thraupidae)"

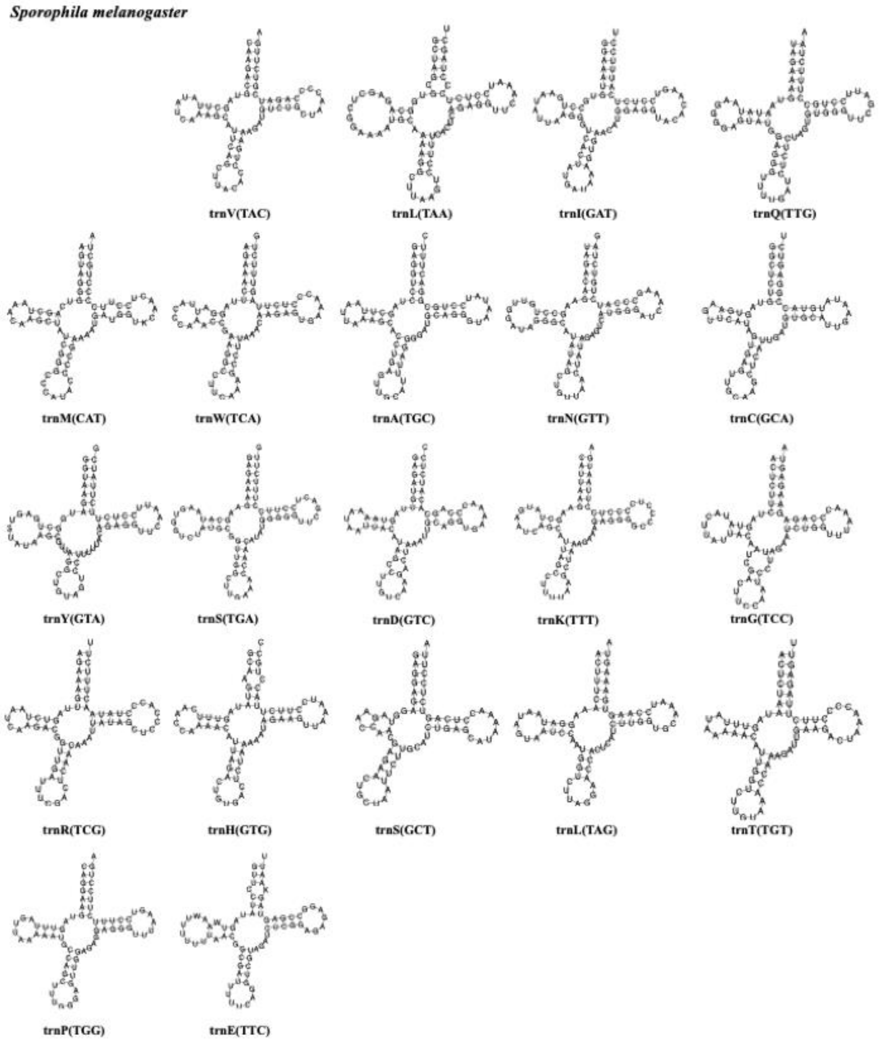


**Figure S5 -** Cover leaf-like structure of the 21 tRNAs present in the mitochondrial genome of *Sporophila melanogaster*.
